# Viral Integration Plays a Minor Role in the Development and Prognostication of Oral Squamous Cell Carcinoma

**DOI:** 10.3390/cancers14215213

**Published:** 2022-10-24

**Authors:** Laveniya Satgunaseelan, Dario Strbenac, Sahithi Tadi, Kevin Nguyen, James Wykes, Carsten E. Palme, Tsu-Hui (Hubert) Low, Jean Y. H. Yang, Jonathan R. Clark, Ruta Gupta

**Affiliations:** 1 Department of Tissue Pathology and Diagnostic Oncology, NSW Health Pathology, Royal Prince Alfred Hospital, Sydney, NSW 2050, Australia; 2 Sydney Medical School, Faculty of Medicine and Health Sciences, The University of Sydney, Sydney, NSW 2050, Australia; 3 School of Mathematics and Statistics, The University of Sydney, Sydney, NSW 2050, Australia; 4 Sydney Head and Neck Cancer Institute, Department of Head and Neck Surgery, Chris O’Brien Lifehouse, Sydney, NSW 2050, Australia; 5 Department of Otolaryngology—Head & Neck Surgery, Faculty of Medicine and Health Sciences, Macquarie University, Sydney, NSW 2109, Australia; 6 Charles Perkins Centre, The University of Sydney, Sydney, NSW 2050, Australia; 7 Royal Prince Alfred Institute of Academic Surgery, Sydney Local Health District, Sydney, NSW 2050, Australia

**Keywords:** viruses, oral squamous cell carcinoma, human papillomavirus (HPV)

## Abstract

**Simple Summary:**

Viruses are well known causes of several human malignancies. Oral squamous cell carcinoma (OSCC) is rising in patients with limited exposure to traditional risk factors, including smoking. As a causative factor of OSCC is yet to be found, our study aimed to identify a virus that may drive this cancer. First, we examined whole genome sequencing data from 28 patients under the age of 50 with limited exposure to carcinogens for viruses. Using viral detection software that screens for >700,000 viruses, we identified one 49 year old male patient with human papillomavirus (HPV). We further validated our findings in 657 patients, using immunohistochemistry and RNA in situ hybridization specific for HPV, and identified 8 (1.2%) male patients with HPV integration. Through a comprehensive search for viruses and evaluation in a large patient OSCC cohort, we demonstrate that viral integration occurs in a minority of male OSCC patients.

**Abstract:**

Viruses are well known drivers of several human malignancies. A causative factor for oral cavity squamous cell carcinoma (OSCC) in patients with limited exposure to traditional risk factors, including tobacco use, is yet to be identified. Our study aimed to comprehensively evaluate the role of viral drivers in OSCC patients with low cumulative exposure to traditional risk factors. Patients under 50 years of age with OSCC, defined using strict anatomic criteria were selected for WGS. The WGS data was interrogated using viral detection tools (Kraken 2 and BLASTN), together examining >700,000 viruses. The findings were further verified using tissue microarrays of OSCC samples using both immunohistochemistry and RNA in situ hybridisation (ISH). 28 patients underwent WGS and comprehensive viral profiling. One 49-year-old male patient with OSCC of the hard palate demonstrated HPV35 integration. 657 cases of OSCC were then evaluated for the presence of HPV integration through immunohistochemistry for p16 and HPV RNA ISH. HPV integration was seen in 8 (1.2%) patients, all middle-aged men with predominant floor of mouth involvement. In summary, a wide-ranging interrogation of >700,000 viruses using OSCC WGS data showed HPV integration in a minority of male OSCC patients and did not carry any prognostic significance.

## 1. Introduction

Viruses have been established as well-known causes of human malignancy affecting a range of organ systems [1]. Virus associated cancers account for 2.2 million cancers worldwide annually [2]. The International Agency for Research on Cancer (IARC) has identified six oncogenic viruses that cause human malignancies: human papillomavirus (HPV), hepatitis B and C viruses (HBV and HCV), Epstein–Barr virus (EBV), human herpesvirus 8 (HHV-8) and human T-cell lymphotropic virus type 1 (HTLV-1) [3].

The demographic and epidemiological features of the affected patient cohorts are often homogenous in virally driven cancers [1]. For example, a large proportion of patients with HBV and HCV associated hepatocellular carcinoma show similar demographic profiles [4], or epidemiologically distinct distributions are observed, as found with EBV in Burkitt lymphoma or nasopharyngeal carcinoma [5]. Furthermore, with the advent of whole genome sequencing (WGS), detection of viruses and evaluation of their role in carcinogenesis has been more extensive than before [6]. For example, the breakpoints of HBV and HPV integration have been established by WGS in hepatocellular [7,8] and and cervical cancer [9] respectively.

Indeed, in the context of head and neck cancer, the oncogenic effects of HPV in oropharyngeal carcinoma are well characterised [10,11], with affected patients being younger without a significant history of tobacco use [12]. This has led to speculation that oral squamous cell carcinoma (OSCC), particularly in young patients who develop OSCC in the absence of cumulative exposure to traditional risk factors such as smoking, may be similarly driven by an oncogenic virus [13,14]. Unfortunately, despite clear anatomical boundaries, many studies fail to distinguish between the oral cavity and the oropharynx when evaluating viral drivers in OSCC [15,16,17]. While earlier studies have cited potential viral drivers of OSCC included HSV and EBV [18], this has not been validated in the current literature [19]. A comprehensive evaluation of the OSCC genome for a viral driver has not been performed to date.

Identifying a potential viral driver for OSCC in patients with low exposure to tobacco will pave the way for preventative public health measures. This study aims at comprehensively investigating the role of viruses in the development of OSCC by utilizing whole genome sequencing (WGS) of OSCC tissues from 28 young patients unlikely to have high cumulative exposure to smoking and using viral databases including >700,000 viruses. Following this, a retrospective cohort of 657 cases of OSCC with complete clinicopathological data and follow-up information was utilized to validate the genomic findings.

## 2. Materials and Methods

### 2.1. Study Cohort

**Figure 1:**
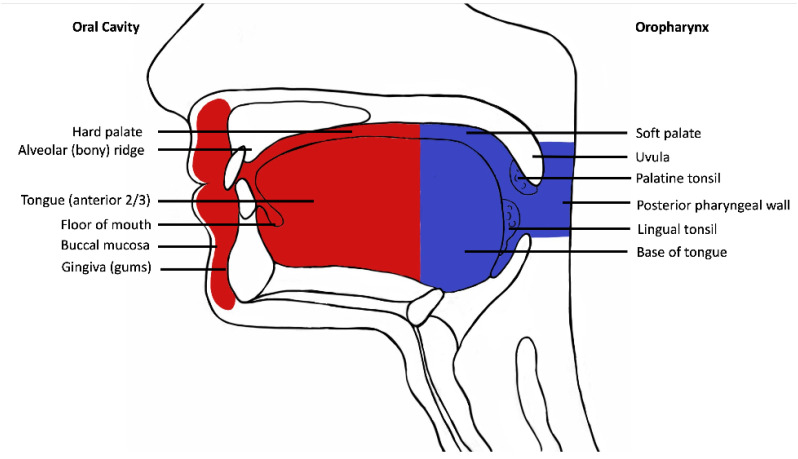
Schematic diagram demonstrating oral cavity anatomy. Oral cavity is depicted in red and oropharynx in blue.

Following institutional human research ethics committee approval (X19-0282/ETH12165), the Sydney Head and Neck Cancer Institute (SHNCI) Biobank was searched for all cases of oral cavity squamous cell carcinoma between 1995 and 2021, with strictly defined anatomic boundaries (Figure 1). The subsites included are oral tongue, floor of mouth, gingiva, palatal mucosa, retromolar trigone, buccal mucosa, and the mucosal lip (Figure 1). All oropharyngeal subsites including the posterior 1/3rd (base) of tongue, tonsils, glossotonsillar folds, and soft palate were excluded. Cases with equivocal subsite descriptions underwent a histological review of their microanatomy, and all resections including tonsillar tissue, circumvallate papillae or respiratory mucosa were excluded.

The SHNCI biobank yielded fresh frozen tissues for nucleic acid extraction and WGS from 28 patients less than 50 years of age to ensure low cumulative exposure to other carcinogens such as smoking. All smoking data is self-reported by the patients. A definition of ‘ever smoker’ was used for ex- and current smokers, whereas ‘never smoker’ was used for a patient who has never smoked in the past.

Formalin fixed paraffin embedded (FFPE) archival tissue was retrieved from 657 cases with complete clinicopathological data, including demographic data, histopathological reports, and survival outcomes for validation. The validation cohort of 657 comprised all cases of OSCC in 1995–2021 who met the inclusion criteria regardless of age or smoking exposure.

### 2.2. Nucleic Acid Extraction and Whole Genome Sequencing (WGS)

Fresh frozen tissue available for 28 OSCC cases underwent nucleic acid extraction using the Qiagen AllPrep Kit (Qiagen, Germantown, MD, USA) as per manufacturer’s instructions. Quantification of DNA and RNA was undertaken using Qubit V2.0 HS assays (Invitrogen, Carlsbad, CA, USA), NanoDrop spectrophotometry (Thermo Scientific, Waltham, MA, USA) and 0.8% agarose gel electrophoresis.

Tumoural and normal DNA underwent pair-ended WGS with a read length of 150 base pairs, and at target depths of 60x and 30x, respectively on the Illumina NovaSeq 6000 platform. Subsequent alignment, variant calling, and variant effect predictions are described in detail on the Sydney Informatics Hub GitHub repository [20,21,22]. Following computational quality checks of tumor purity, 28 OSCC cases were available for analysis.

### 2.3. Viral Insertion Detection from WGS Data

Structural variants were identified using GRIDSS 2 [23] which also assembles reads into longer contigs at breakends. Only variants with a PASS value for FILTER were retained. The assembled sequences were annotated using Kraken 2 using its custom database [24,25], that includes the Joint Genome Institute’s Integrated Microbial Genomes and Microbiomes (IMG) database of over 700,000 viruses [26]. In addition, the NCBI viral genomes resource was also utilized (https://www.ncbi.nlm.nih.gov/genome/viruses/about/assemblies/, accessed on 15 March 2022) [27]. Any viruses identified were further verified by annotating the assembled sequence using NCBI BLASTN [28] and its non-redundant Nucleotide database using the discontiguous megablast search strategy [29]. Sequences purported to be viral insertions by Kraken 2 were considered to be valid if BLASTN also reported the same virus and at least 97% of bases matched the database sequence [24].

### 2.4. Mutation Signature Detection

Catalogue of Somatic Mutations in Cancer (COSMIC) mutational signature analysis was performed using COSMIC v3 [30], with the Mutational Patterns Bioconductor package [31]. Particular attention was paid to the activation-induced cytidine deaminase/apolipoprotein B mRNA-editing catalytic polypeptide-like (AID/APOBEC) related signatures, single base substitution (SBS) mutation signatures 2 and 13, given their relationship to viral infection [30]. In addition, three newly identified mutation signatures associated with the with HPV16 infection were also investigated [32].

### 2.5. Histopathological Review and Tissue Microarray Construction

All 657 cases underwent histopathological review. Regions of high tumour cellularity were selected for sampling. Two cores of 1.0 mm in diameter were obtained using the Beecher Manual Tissue Microarrayer (Model MTA-1; Diagnostic Technology, Australia), avoiding areas with necrosis, haemorrhage, keratinisation or surgical diathermy artefact.

### 2.6. p16 Immunohistochemistry

3 μm thickness tissue sections were cut from the TMAs onto charged SuperFrost Ultra Plus slides (Menzel-Glaser, Thermo Fisher Scientific, Bremen, Germany). p16 immunohistochemical staining was undertaken on the Leica-Bond III autostainer (Leica Microsystems, Wetzlar, Germany). Pretreatment ER2 (epitope retrieval buffer) was performed for 30 min, after which heat-induced epitope retrieval with an EDTA based buffer, pH 9 (Leica Microsystems). Tissue sections were incubated with a Mouse anti-Human p16 (E6H4) primary antibody (CINtec Histology Kit; Roche Ventana, Oro Valley, AZ, USA). p16 protein detection was performed using Biotin-free Polymer Refine Detection Kit (Leica Microsystems).

p16 staining of any intensity was recorded. However, a designation of ‘strong p16 staining’ was documented in those cases where there was strong diffuse nuclear and cytoplasmic staining in greater than 70% of tumor cells [33,34].

### 2.7. HPV In Situ Hybridisation (ISH)

RNAscope HPV-16/18 and RNAscope HPV-31, HPV-33, and HPV-35 were performed using Leica Bond III automated staining platform. Heat-mediated antigen retrieval (95 °C) was used at pH 9 for 15 min. Bond RNAscope Protease was used for 15 min at 40 °C. Each of the probes (RNAscope 2.5 LS Probe HPV16/18, HPV-31, HPV-33 and HPV-35) was hybridized for 120 min at 42 °C. Bond RNAscope Detection Reagents were used with the standard Bond III RNAscope DAB ISH protocol.

HPV-positive status by RNA ISH was determined by the presence of cytoplasmic punctate dot-like staining within the tumor cells only. Control tissue using cervical intraepithelial neoplasia (CIN3) was included for both p16 immunohistochemical staining and HPV RNA ISH.

### 2.8. Statistical Analysis

Disease specific survival was calculated from the date of surgery to death from OSCC or date of last follow-up from OSCC. Disease-free survival was calculated from the date of surgery to the date of recurrence or death. Overall survival was calculated from the date of surgery to the date of death or date of last follow-up. Survival was calculated using the Kaplan–Meier method and comparisons were made using the log-rank test.

## 3. Results

WGS and interrogation for viral integration were performed on 28 cases, including 16 men and 12 women. The median depth of coverage for tumor tissue was 85X (range 64X–140X) and for matched normal tissue was 45X (range 16X–75X).

From the 28 cases, Kraken 2 detected three viral insertions on examination of the WGS data: (1) HPV35, (2) Megavirus chilensis, and (3) Pandoravirus neocaledonia. Of these three viral inserts, HPV35, demonstrated a 100% match using BLASTN. The other two viral insets showed no viral taxonomic homology via BLASTN, and therefore were considered to have inadequate evidence of viral integration.

**Figure 2:**
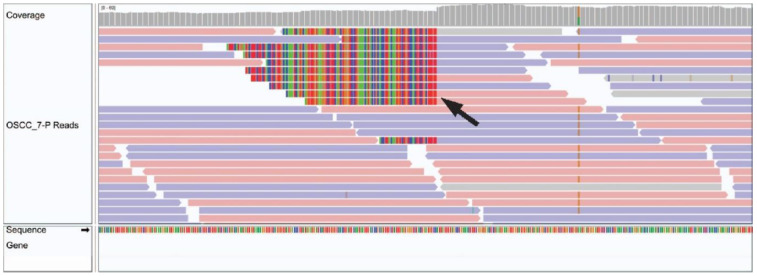
Integrated Genome Viewer image of HPV integration in OSCC tumour genomic sample. A black arrow demonstrates a genomic breakend of an HPV viral insertion.

Integration of HPV35 was observed in chromosome 10 of a 49 year old male patient with a hard palate OSCC (Figure 2). No other viruses from the custom Kraken 2 database, including well recognized oncogenic viruses such as HPV, HBV, HCV, EBV, HHV-8 and HTLV-1 or novel viruses from the 700,000 viruses included in the NCBI viral genome database, were identified via BLASTN in the remaining 27 cases.

### 3.1. APOBEC Signatures Are Neither Specific nor Sensitive for HPV Detection in OSCC

**Figure 3:**
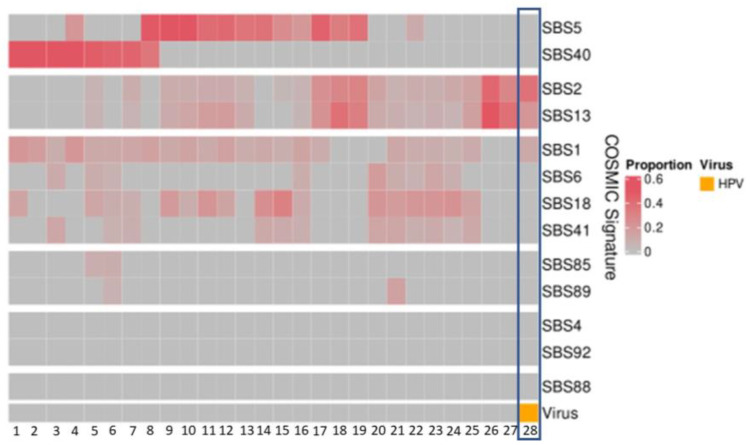
Mutational signatures (COSMIC3) of patient cohort (*n* = 28). The HPV-positive patient (patient 28 with HPV35) demonstrates high proportions of SBS2 and SBS13.

The APOBEC-related mutation signatures, SBS signatures 2 and 13, were detected at proportions between 0.04 and 0.57 in 21 patients (Figure 3). In the patient with HPV35, the APOBEC mutation pattern was present at proportions of 0.38 (SBS2) and 0.21 (SBS13) (Figure 3).

Three newly identified mutation signatures associated with HPV in cervical cancer [32] were also examined within our patient cohort and none were identified.

### 3.2. HPV-Positive OSCC Is Genomically Distinct from HPV-Negative OSCC

**Figure 4:**
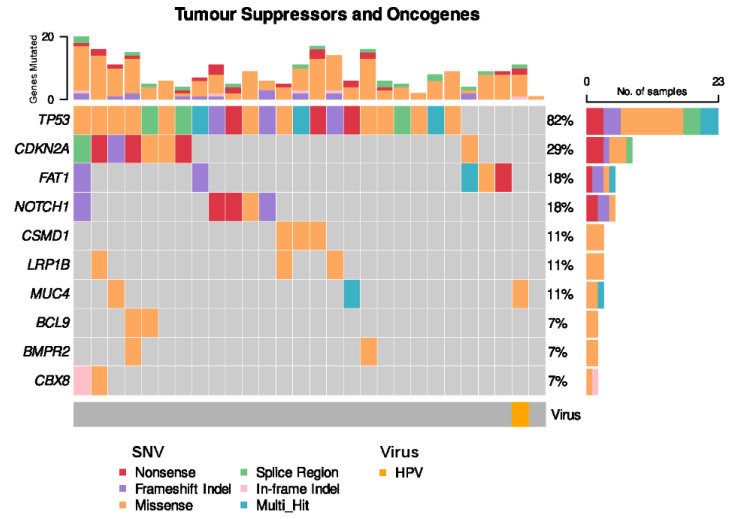
Frequent single nucleotide variants (SNVs) in patient cohort.

Somatic *TP53* non-synonymous single nucleotide variants (SNVs) were present in the vast majority of OSCC cases (22 of 28 cases, 82%) (Figure 4). *CDKN2A* SNVs were seen in 8 cases (29%). The solitary HPV-positive case of OSCC harboured neither a *TP53* nor *CDKN2A* alteration, but harboured a somatic missense SNV in *MUC4*, which plays a role in modulating cell apoptosis in epithelial cancers [35].

### 3.3. Investigating the Presence of HPV in a Larger OSCC Cohort

The finding of HPV in OSCC has been reported in small cohorts or cohorts that include patients with both oral and oropharyngeal SCC. Further, the emphasis has generally been on HPV16/18. Hence, we sought to further investigate the genomic finding of HPV35 and multiple high risk HPV subtypes including HPV 16, 18, 31, 33 and 35 in OSCC using an anatomically well-defined cohort of 657 patients.

**Figure 5:**
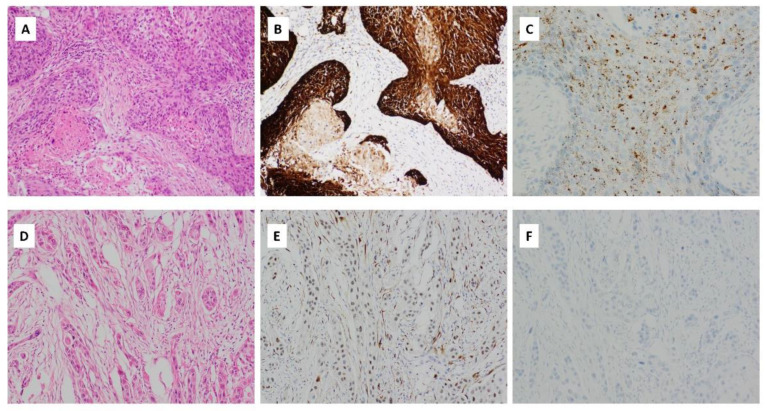
(**A**) Haematoxylin and eosin (H&E) stained sections of OSCC with HPV integration. There are expanded nests of keratinocytes with scanty cytoplasm and angulated hyperchromatic nuclei. Comedonecrosis is seen. There is scanty keratinization at the periphery of the nests (magnification ×400); (**B**) p16 immunohistochemical staining, showing strong, cytoplasmic and nuclear block-like positive staining in >75% of the cells (magnification ×400); (**C**) HPV RNA ISH demonstrating punctate tumour cell staining (magnification ×400); (**D**) H&E stained section of OSCC with weak/patchy p16 immunohistochemical staining (magnification ×400); (**E**) p16 immunohistochemical staining with patchy weak p16 staining considered negative for p16 (magnification ×400); (**F**) HPV RNA ISH demonstrating lack of HPV integration (magnification ×400).

The 657 OSCC patients (Figure 5) included 368 men and 289 women with a median age of 65 years. All patients were treated with surgical resection with curative intent. Adjuvant radiotherapy was required in 258 patients and chemotherapy in 70 patients (Table 1). Complete follow up of a median of 2 years was available (range 0.01 to 18.5 years). 136 patients developed recurrence and/or metastases and 223 patients died of OSCC.

Immunohistochemistry for p16 and in situ hybridization for HPV subtypes 16, 18, 31, 33, 35 mRNA was performed on all 657 patients.

### 3.4. Patterns of p16 Immunohistochemical Staining in Oral Cavity Squamous Cell Carcinoma

p16 immunohistochemical staining of any intensity was seen in 112 (17%) of OSCC samples (Figure 5B,E, Table 2). Demographic characteristics, anatomical site of disease, pathological TNM staging, and adjuvant treatment were similar between patients showing p16 immunostaining of any intensity and p16 immunonegative patients.

Strong p16 immunohistochemical staining with block cytoplasmic and nuclear staining in >70% of the tumor cells (as defined by the College of American Pathologists [34]) was seen in 45 (6.8%) cases (Figure 5B, Table 2). Most of these involved the tongue (17 cases, 38%) and floor of mouth (14 cases, 31%). The majority of cases were moderately keratinising differentiated SCCs (*n* = 31, 69%). 18 cases demonstrated lymphovascular invasion (40%) and 19 cases perineural invasion (42.2%). The clinicopathological profile was otherwise similar to OSCC with any intensity of p16 immunohistochemical staining and those lacking p16 immunostaining (Figure 5E, Table 2).

### 3.5. HPV Integration Is Rare in OSCC

Eight cases of HPV integration were detected by mRNA ISH (1.2%; Figure 5C, Table 3). All cases were male, with an age range between 49 and 69 years (median 60 years). Five cases (62.5%) involved the floor of the mouth and the remaining three involved tongue, buccal mucosa, and hard palate, respectively. Involvement by HPV genotypes HPV16/18 (*n* = 6), HPV31 (*n* = 0), HPV33 (*n* = 1) and HPV35 (*n* = 1) were seen. The tumors demonstrated morphologic features similar to those observed in oropharyngeal SCC, with expanded nests of keratinocytes, however without lymphoid stroma. The cells showed scanty cytoplasm and angulated, hyperchromatic nuclei. Most nests showed comedonecrosis and scanty keratinisation at the periphery of the nests (Figure 5A). The adjacent mucosa showed squamous cell carcinoma in situ as well as extension of carcinoma in situ along minor salivary gland ducts. Four cases harboured lymphovascular invasion (50%), and three cases perineural invasion (37.5%). 50% of HPV-positive cases received adjuvant radiotherapy, as compared to HPV-negative cases, where 11% underwent adjuvant radio- and/or chemotherapy (Table 3).

### 3.6. Use of p16 Immunohistochemistry as a Surrogate Marker of HPV Integration Has Low Positive Predictive Value in OSCC

Strong block p16 immunostaining was seen in 45 (6.8%) OSCC cases, with eight (17.8%) of these showing HPV integration. All HPV-positive cases demonstrated strong p16 immunohistochemical staining. All p16-negative cases were HPV-negative by RNA ISH. While this results in high sensitivity (100%), specificity (94.3%) and negative predictive value (100%), the positive predictive value of p16 immunohistochemistry as an indicator of HPV integration in OSCC was only 17% even when block cytoplasmic and nuclear staining was considered.

### 3.7. p16 Immunohistochemistry Is Not a Surrogate of CDKN2A Deletion in OSCC

Amongst the 28 patients with WGS data, seven showed p16 immunostaining of any intensity (25%) and four cases demonstrated strong p16 staining (14.2%). None of the cases with p16 immunoreactivity, regardless of intensity, harboured a *CDKN2A/B* deletion or other alteration.

### 3.8. p16 Status and HPV Integration in OSCC Are Not Related to Survival

**Figure 6:**
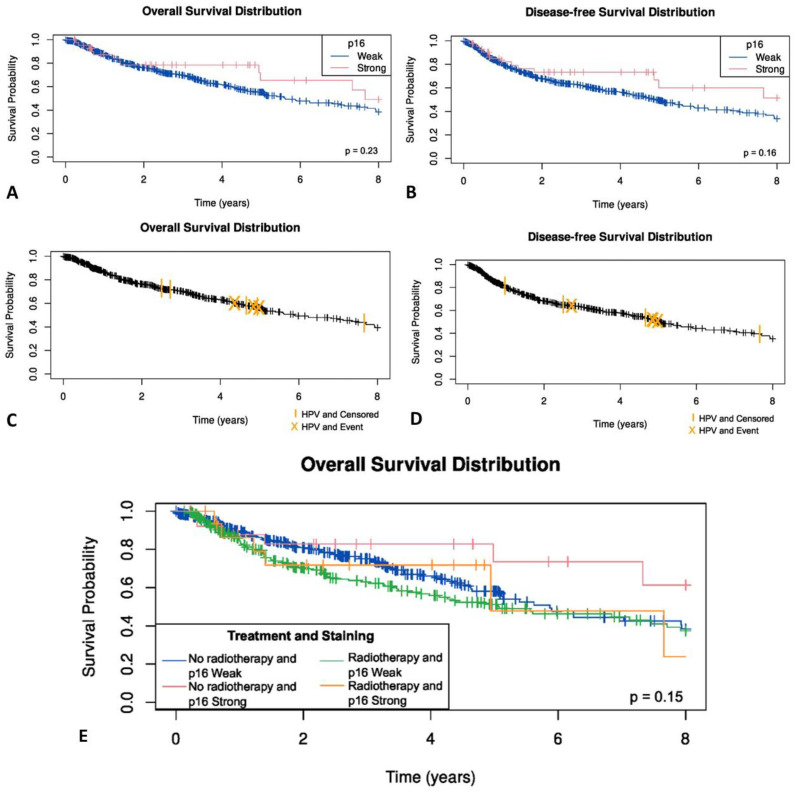
(**A**) Overall survival in OSCC by p16 status; (**B**) disease free survival by p16 status; (**C**) Overall survival in OSCC by HPV status; (**D**) disease free survival by HPV status; (**E**) Kaplan–Meier survival curves by radiotherapy and p16 status in OSCC patients.

No statistically significant difference was observed between p16-negative OSCC patients and those demonstrating weak, or block cytoplasmic and nuclear p16 immunostaining for overall survival (*p* = 0.35) (Figure 6A), disease specific status (*p* = 0.52) or disease free survival (*p* = 0.61) (Figure 6B). Similarly, no significant difference between HPV-positive and HPV-negative OSCC patients was seen for overall survival (*p* = 0.49) (Figure 6C), disease specific status (*p* = 0.25) or disease free survival (*p* = 0.81) (Figure 6D).

The effect of radiotherapy was examined. In those patients who had radiotherapy, no statistically significant difference was observed between p16-negative OSCC patients and those demonstrating weak, or blocked cytoplasmic and nuclear p16 immunostaining (*p* = 0.32) (Figure 6E). As only eight OSCC patients were HPV-positive, the effect of radiotherapy on survival in HPV-positive OSCC was not examined due to the limited number of events.

## 4. Discussion

Viruses are a well-established cause of human cancer, with nearly a fifth of cancers associated with viral drivers across different body systems [1]. The six oncogenic viruses as identified by the IARC have distinct demographic and epidemiological profiles [1]. For example, HHV8-driven Kaposi sarcoma forms a major burden of disease in those areas where HHV8 is endemic, particularly affecting males of Mediterranean origin, or in those with human immunodeficiency virus (HIV) [36]. Similarly, Burkitt lymphoma was initially identified as a childhood haematolymphoid malignancy with a specific geographic distribution in equatorial Africa [37]. This led to the hypothesis that an underlying infective agent restricted to this region was a likely cause, and EBV was subsequently identified as the oncogenic driver [38]. Given the rising incidence of OSCC in young patients lacking significant exposure to both tobacco and alcohol use [14], a viral etiology warrants investigation in this cohort.

WGS followed by interrogation with a 700,000 virus strong comprehensive NCBI database was performed to explore potential viral integration in OSCC. Two complementary approaches were implemented to ensure robust results. Initially, the Kraken 2—*k*-mer mapping method was coupled with confirmation through BLASTN—full sequence alignment method. The utilisation of WGS data to detect viral integration is reliant on establishing homology between sequencing reads and known reference viral sequences [39]. This can be determined by either using a portion of the sequencing read and finding an exact match in the viral sequence (*k*-mers mapping), or full alignment of a sequencing read [39]. Kraken 2 uses *k*-mer mapping to provide a precise method of assigning viral taxonomy with large volumes of sequencing data against numerous vast viral databases including both the JGI and NCBI databases which include over 700,000 viruses [24,26]. In addition, Kraken 2 has been shown to have high precision metrics, including high specificity, in benchmarking studies [39,40]. Complimenting this approach, BLASTN has shown high sensitivity for the detection of viral reads, with full sequences assessed against a viral reference genome [39]. Thus, employing both Kraken 2 and BLASTN, we undertook a comprehensive assessment for viruses in 28 OSCC patients with low cumulative mutation burden from traditional risk factors and identified HPV35 in a single patient. This tumor lacked a *TP53* mutation, which was otherwise observed in 82% of the cohort. It was also the only case to harbour a *MUC4* somatic missense SNV. *MUC4* encodes the protein MUC4, an epithelial mucin, with overexpression of the protein seen in many epithelial cancers [41]. *MUC4* mutations have been described in HPV-positive SCC in exome sequencing studies and have been shown to be an important regulator of cell death in epithelial cancers [35,42]. In OSCC, *MUC4* overexpression assessed by immunohistochemistry has been associated with poor prognosis [43,44], with a study of a p53 null murine OSCC cell line showing high mutation rates of *MUC4* [45].

Amongst the patients, with WGS data, 21 patients showed the presence of COSMIC mutational signatures (SBS 2 and SBS13). The newly described mutational signatures in a study of HPV16-driven cervical cancer were also not observed in our cohort [32]. Interestingly, no other viruses were found in these 21 patients, despite COSMIC mutational signatures 2 and 13 attributed to the AID/APOBEC family of cytidine deaminases, which are typically activated in viral infection [30]. The absence of viral integration in the presence of SBS2 and SBS13 is not clearly described [46]. Our results would indicate that, AID/APOBEC mutation signatures do not appear to be a specific indicator of the presence of viral integration in malignancies with low prevalence of viral integration such as OSCC.

The finding of one patient with integration of a rare HPV subtype prompted us to examine a large cohort of 657 OSCC patients. HPV integration by RNA ISH for multiple high risk HPV including rare subtypes such as 31, 33 and 35 in addition to the common HPV 16/18 were examined and demonstrated a very low incidence of HPV integration in 8 (1.2%) patients. This is in contrast to other studies which report a highly variable prevalence, ranging from 2.2% to 61% [15,47]. A variety of methods including p16 immunohistochemistry, HPV DNA in situ hybridisation (ISH), HPV reverse-transcription PCR (RT-PCR) and RNA ISH [16,48,49] have been used in the detection of HPV-integration in head and neck SCC. The numerous available methods and use of inappropriate anatomic terminology make comparisons between studies difficult [48,49,50,51]. Similar to our findings, a recent study by Nauta et al. [47], found an incidence of 2.2% of HPV integration in OSCC using an HPV DNA RT-PCR assay. RNA ISH was used in our study as it is a practical and sensitive detection method for HPV under light microscopy, with similar sensitivity to RT-PCR methods and improved sensitivity as compared to DNA ISH [52,53,54]. The results of our study utilising comprehensive methods of viral detection suggest that in the vast majority of patients, OSCC is not virally driven and has a very low incidence of 1.2% of HPV integration. Thus, it is becoming increasingly important that appropriate anatomic boundaries and terminology are applied in distinguishing tonsillar tissue of the oropharynx where HPV is now the predominant driver of malignancy from the oral cavity.

Although widely used in clinical practice for oropharyngeal and cervical SCC, p16 immunohistochemistry is not a reliable surrogate marker of HPV integration in OSCC where the prevalence of HPV integration is low [55]. Our study determined that while p16 immunohistochemistry has high sensitivity, specificity and negative predictive value, the positive predictive value of p16 for the detection of HPV in OSCC was only 17%, lower than that found by Nauta et al. (45.7%) [47]. It is a well-recognized fact that cell cycle abrogation in the p15(INK4b)/p16(INK4a)-cyclinD/CDK4-RB1-mediated pathway can lead to strong nuclear and cytoplasmic p16 immunostaining of malignant cells [56]. Such staining is well recognized in a range of unrelated malignancies including cutaneous squamous cell carcinoma, melanoma, neuroendocrine carcinomas and lymphomas [54]. Thus, it is critical that p16 immunostaining is only performed in the appropriate clinical and morphologic context and accompanied by appropriate squamous lineage specific immunohistochemistry such as p40 or CK5/6 to avoid misdiagnoses (48).

It is important to note that all eight p16/HPV-positive OSCC patients were male, between 49 and 69 years, with the majority (62.5%) arising from the floor of the mouth, as has also been recently described by Lewis Jr. et al. [57]. The HPV positive OSCC showed morphologic features similar to that observed in HPV associated oropharyngeal SCC. Two studies evaluating HPV integration in OSCC demonstrate a similar gender and subsite predilection, with HPV-positive OSCCs arising on the floor of mouth in 21% [58] and 37.5% [59] of cases, respectively, and an association with male gender [59]. Thus, this cohort of 657 anatomically well-defined cases of OSCC highlights that HPV integration plays a minor role in OSCC. While a range of rare high risk HPV subtypes was tested in this study, it is possible that some of the strongly p16 positive cases in this cohort may harbour extremely rare HPV subtypes [60]. However, neither the detection of p16 nor HPV in OSCC correlated with survival outcomes, as no survival differences were observed between p16-positive and p16-negative cases in this cohort.

HPV/p16-positive oropharyngeal SCC is associated with improved prognosis, regardless of the modality of treatment [61]. Studies that combine all non-oropharyngeal head and neck sites, as opposed to examining OSCC in isolation, propose that HPV and p16 have a similar prognostic role in non-oropharyngeal SCC [62,63]. However, similar to our findings, those studies which examined the role of p16 and HPV in OSCC alone showed no prognostic value using either biomarker [15,47,58].

A main limitation of this study is the availability of WGS data in 28 patients only. This study involving WGS and comprehensive bioinformatic analyses in 28 patients coupled with validation using a cohort of 657 cases with detailed clinicopathologic data is extremely resource intensive [64]. While the incidence of OSCC is rising in patients younger than 50 years, this is a relatively recent trend [14]. Of note, The Cancer Genome Atlas (TCGA) in its multi-institutional efforts includes only 10 patients younger of 50 years of age [65]. Additionally, WGS needs high quality tumour DNA requiring access to a functional biobank with streamlined protocols for collection of tissues immediately after resection. WGS is expensive and the analyses of the vast data generated requires specific expertise in bioinformatic analyses. Additionally, a well maintained complete clinicopathologic database with associated archival tissues is a valuable resource. Surmounting these difficulties is a unique strength of the current study. The findings of this exhaustive analyses demonstrate that testing for HPV or p16 is not essential for management of OSCC patients.

## 5. Conclusions

In conclusion, by using WGS techniques and interrogation of a large viral dataset to examine for the broadest range of potential viruses, we demonstrate that the vast majority of OSCC is not associated with viral integration, even in patients with low cumulative exposure to known carcinogens such as tobacco. Furthermore, HPV integration occurs rarely in OSCC, and where it does occur, the HPV-positive OSCC case has the profile of a middle-aged male with a floor of mouth SCC, morphologically resembling oropharyngeal SCC. Resource intensive detection of p16 or HPV in OSCC is unlikely to have any clinical significance in OSCC.

## Figures and Tables

**Table 1:** Cohort summary.

**N**	657
**Age (years, median, range)**	65 (18–95)
**Sex (M:F)**	368:289
**Sites**	
Oral tongue	294
Floor of mouth	125
Retromolar trigone	38
Buccal mucosa	67
Alveolar	97
Hard palate	18
Mucosal lip	18
**pT stage**	
1	231
2	184
3	67
4	175
**pN stage**	
0	399
1	74
2	166
3	18
**Adjuvant treatment**	
Radiotherapy	258
Chemotherapy	70
Radiation and chemotherapy	69

**Table 2:** Demographics by p16 immunohistochemical status.

N	p16 Strong Positive (*n* = 45)	p16 Positive (Weak or Patchy) (*n* = 67)	p16 Negative (*n* = 547)
**Age (years, median, range)**	65 (23–94)	65 (33–95)	66 (18–94)
**Sex (M:F)**	13:32	37:30	300:245
**Sites (*n*, %)**			
Oral tongue	17 (38%)	33 (45%)	244 (45%)
Floor of mouth	14 (31%)	9 (21%)	102 (19%)
Retromolar trigone	0	3 (3%)	35 (6%)
Buccal mucosa	7 (16%)	4 (10%)	56 (10%)
Alveolar	6 (13%)	10 (14%)	81 (15%)
Hard palate	1 (2%)	4 (4%)	13 (2%)
Lip	0	4 (3%)	14 (2%)
**T stage (*n*, %)**			
1	17 (38%)	32 (48%)	182 (33%)
2	14 (31%)	17 (25%)	153 (28%)
3	6 (13%)	5 (7%)	56 (10%)
4	8 (18%)	14 (21%)	153 (28%)
***n* stage (*n*, %)**			
0	26 (58%)	49 (73%)	324 (59%)
1	5 (11%)	3 (5%)	66 (12%)
2	13 (29%)	13 (19%)	140 (26%)
3	1 (2%)	1 (2%)	16 (3%)
**Adjuvant treatment (*n*, %)**			
Radiotherapy	16 (36%)	18 (27%)	224 (41%)
Chemotherapy	5 (11%)	4 (6%)	61 (11%)
Radiation-/chemotherapy	5 (11%)	4 (6%)	60 (11%)

**Table 3:** Demographics by HPV status.

N	HPV+ (*n* = 8) HPV16/18 (*n* = 6) HPV33 (*n* = 1) HPV35 (*n* = 1)	HPV− (*n* = 649)
**Age (years, median, range)**	60 (49–69)	65 (18–95)
**Sex (M:F)**	8:0	360:289
**Sites**		
Oral tongue	1 (13%)	293 (45%)
Floor of mouth	5 (63%)	120 (18%)
Retromolar trigone	0	38 (6%)
Buccal mucosa	1 (13%)	66 (10%)
Alveolar	0	97 (15%)
Hard palate	1 (13%)	17 (3%)
Lip	0	18 (3%)
**pT stage**		
1	2 (25%)	229 (35%)
2	2 (25%)	182 (28%)
3	1 (13%)	66 (10%)
4	3 (37%)	172 (26%)
**pN stage**		
0	0 (0%)	399 (62%)
1	1 (13%)	73 (10%)
2	3 (37%)	163 (25%)
3	0	18 (3%)
**Adjuvant treatment**		
Radiotherapy	4 (50%)	254 (39%)
Chemotherapy	0	70 (11%)
Radio-/chemotherapy	0	69 (11%)

## Data Availability

The data presented in this study are available on request from the corresponding author. The data are not publicly available due to ethics and privacy restrictions.
